# Supplementary material for: The Gut Microbiome in the First One Thousand Days of Neurodevelopment: A Systematic Review from the Microbiome Perspective
Source: Microorganisms. 2024 Feb 20;12(3):424. doi: 10.3390/microorganisms12030424 (PMC10972197; doi:10.3390/microorganisms12030424)
Supplement: Supplementary file 1 [file microorganisms-12-00424-s001.zip › microorganisms-2820872-supplementary.pdf]

Current knowledge

Table S1. Summary of epidemiological studies investigating the association between preterm infants' gut microbiome and neurodevelopment.

Publication Author(s) Year	Study design (Year) Setting	Population (N)	Microbiome		Neurodevelopment outcomes		Covariates	Key Findings
			**Method**	**Age**	**Instruments**	**Age**		
Beghetti et al. 2021	Longitudinal (2014?) Italy	Age < 32 weeks (N = 27)	16S rRNA	Feces at: 1, 4, 7, and 30 days	(GMDS)	24-month corrected age	Not described	• Beta-diversity significantly different between infants with normal and neurodevelopmental impairment (NI) ($p \leq 0.05$).<br>• NI: increased *Bifidobacteriacea* at day 7 ($p = 0.05$).<br>• *Bifidobacterium* (at day 30) directly correlated with 24-month GQ score (p= 0.01, tau = 0.449). Major represented species were *B. longum* and *B. breve* (both not found in the gut microbiota with NI).
Seki et al. 2021	Longitudinal (2017-2019) Austria	Age < 28 weeks (N = 60)	16S rRNA (V4 region; qPCR (primer pair 515F and 806R).	Days: 3 and 7. Weeks: 2, 4, 6, 8, 10 and 12	Cerebral oxygenation, Cranial MRI	Days: 3 and 7. Weeks: 2, 4, 6, 8, 10 and 12	Not adjusted	• *Klebsiella* was more abundant at 4 weeks post-delivery in infants with severe brain injury ($p < 0.01$).<br>• *Bifidobacterium* sp. at 6 weeks and *Finegoldia* sp. were increased in infants with severe brain injury ($p < 0.05$).<br>• Increased *Enterococcus*, *Escherichia-Shigella*, and *Streptococcus* (p = 0.008, 0.028, and 0.045, respectively) 3 days post-delivery, and *Enterococcus* 2 weeks post-delivery ($p < 0.01$) in infants without severe brain injuries.

								• *Klebsiella pneumoniae* ASV_c07_vnh_xuf at 4 weeks was significantly associated with severe brain injury (p < 0.05).
Oliphant et al. 2021	Longitudinal (2010-2018) USA	Age < 34 weeks (N = 58)	16S rRNA	27, 30, 33, 36 weeks	Head circumference growth (HCG)	From birth to 36 weeks Pos-menstrual age (PMA)	Delivery mode, and patient (random effect)	• Beta-diversity was distinct between infants with appropriate HCG trajectories versus any suboptimal HCG trajectories (SHCGT) (p = .001). Higher *Bacteroidota* (p = .01) and *Lachnospiraceae* (p < .05) were identified as potential biomarkers of AHCGT (adequate), a further reduction in *Actinobacteriota* (−0.78, p < .01), leading to moderately to severely SHCGT.<br>• Higher *Corynebacterium* (1.76, p = .001) and lower *Bacteroides* in SHCGT (−1.78, p = .009).<br>• Lower *Faecalibacterium prausnitzii* (p < .05), *Megasphaeraceae* (p < .05) and *Negativicoccaceae* (p < .01) in SHCGT.
Rozé et al. 2020	Longitudinal (2011-2018) France	Age < 31 weeks. (N = 577)	16S rRNA (V3–V4 region)	Week 4	ASQ-3	2 years	Gestational age; Maternal age, country of birth, education, pre-pregnancy body mass index. Sex, birth weight, c-section, surfactant use, skin-to-skin contact, breastfeeding	• Clusters with dominance of *Enterococcus* and *Staphylococcus* were significantly associated with 2-year non-optimal outcome (death or ASQ-3 score <185) (p = 0.03).

GMDS: Griffiths Mental Development Scale; MRI: Magnetic Resonance Imaging; ASQ-3: Ages and Stages Questionnaire.

Table S2. Summary of epidemiological studies investigating the association between term infants' gut microbiome and neurodevelopment.

**Publication Author (s) Year**	**Study design (Year) Setting**	**Population (N)**	**Microbiome**		**Neurodevelopment outcomes**		**Covariates**	**Key Findings**
			**Method**	**Age**	**Instruments**	**Age**		
Guzzardi *et al.* 2022	Longitudinal (2011–2014) Italy	All born at term (weeks not informed) (N = 90)	16S rRNA (V3-V4 region)	Meconium (n = 79) and feces 3, 6, 12, and 36 months (n = 40, 47, 37, 21)	GMDS	6–24 and 36-60 months	Delivery mode and sex	• Meconium *Bifidobacterium* and *Veillonella* abundances were directly associated with practical reasoning scores at 60 months; p-value not informed.<br>• At 60 months, GMDS total cognition score ($p < 0.05$) was associated with bacterial composition in meconium, mainly determined by *Bifidobacterium* abundance.<br>• Alpha and beta diversity findings were only descriptive.
Tamana *et al.* 2021	Longitudinal (2009-2012) Canada	Age >35 weeks (N = 405)	16S rRNA (V4 region)	4 and 12 months	BSID	1 year and 2 years	Birth mode, sex, maternal ethnicity, older sibling, breastfeeding status at 6 months, family income, maternal	• *Bacteroidetes*-dominant cluster was associated with higher scores for cognitive (β= 4.8), language (β= 4.2), and motor (β= 3.1) development at age 2. All $q < 0.05$.<br>• Boy infants with a *Bacteroidetes*-dominant microbiota (12 months) had more favorable cognitive (β = 5.9, q = 0.06) and language (β = 7.9, $q < 0.001$) development at age 2.<br>• Different species of *Bacteroides* including *B. fragilis*, *B. uniformis*, and unclassified *Bacteroides* were directly associated with cognitive and language development (all $q < 0.05$).

							overweight, and age at sampling.	• Enhanced sphingolipid synthesis and metabolism, and competition between *Bacteroides* and *Streptococcus* were characteristic of a Bacteroidetes-dominant gut microbiota.
Carlson *et al.* 2021	Longitudinal (Year not informed) USA	Age >37 weeks (N = 34)	16S rRNA and Functional prediction (PICRUSt pipeline)	1 month and 1 year	Strange Situation; and Neuroimaging	1 month and 1 year	Age at scan and sex	• 1-month alpha-diversity inversely associated with 1-yo fear behavior score ($p < 0.001$, $n = 19$).<br>• 1-month Weighted Unifrac inversely associated with 1-year medial prefrontal cortex volume ($p < 0.05$, $n = 14$).<br>• 1-month *Streptococcus* inversely associated with 1-month amygdala ($q < 0.05$), hippocampus ($q = 0.132$) and pre-frontal cortex volumes ($q = 0.123$).<br>• 1-month *Bacteroides* directly associated with 1-month amygdala volume ($q = 0.074$).<br>• 1-month *Lachnospiraceae* directly associated with 1-month PFC volume ($q = 0.123$).<br>• 1-month *Veillonella* directly associated with 1-year PFC volume ($q = 0.052$).<br>• 1-month *Enterobacteriaceae* inversely associated with 1-year PFC volume ($q = 0.052$).<br>• 1-year *Dialister* directly associated with non-social fear behavior (Vocal Distress) ($q = 0.143$).<br>• 1-year beta-diversity inversely associated with 1-year non-social fear behavior ($p < 0.001$) and amygdala volume ($p < 0.05$).
Carlson *et al.* 2018	Longitudinal (Year not informed)	Age ~37 weeks (N = 89)	16S rRNA (V1-V2 region)	1 year	MS, and structural MRI	1 and 2 years	C-section, older siblings, paternal	• Mullen scores (receptive and expressive language) at age 2 differed between clusters with *Bacteroides* dominance showing the highest performance and *Faecalibacterium*-dominant cluster showing the

	USA						ethnicity, breastfeeding, sex, maternal education, paternal age and ethnicity, twin status, income.	lowest performance ($p < 0.01$).<br>• Higher alpha diversity associated with lower scores on the overall composite score, visual reception scale, and expressive language scale at age 2 ($p < 0.05$).<br>• Gut microbiome had minimal effects on brain volumes at 1 and 2 years of age.
Gao *et al*. 2018	Longitudinal (Year not informed) USA	Age ~39 weeks (N = 39)	16S rRNA (V1-V2 region)	1 year	MS and Resting-state functional MRI	1 and 2 years	Paternal ethnicity and age, older siblings, sex, maternal education, twin status, and income	• Alpha-diversity and left amygdala functional connectivity (Faith: $r = -0.55$, $p < 0.001$; Shan: $r = -0.36$, $p < 0.05$).<br>• Connectivity between anterior cingulate cortex and right anterior insula associated with Chao1 ($r = -0.54$, $p < 0.001$) and Shannon ($r = -0.42$, $p < 0.01$).<br>• Functional connectivity between supplemental motor area and left parietal cortex associated with alpha diversity ($r = 0.41$, $p < 0.01$).
Ou *et al*. 2022	Longitudinal (Year not informed) Netherlands	Full-term (~40 weeks) (N = 193)	16S rRNA (V4 region)	1, 3 and 4 months, and 6 and 10 years.	CBCL	1, 3 and 4 months, and 6 and 10 years.	Child age, delivery mode, and breastfeeding.	• Bacterial clusters (infancy and childhood) were not associated with behavior.
Acuña	Cross-	Full-	16S rRNA	18 months	BSID	18	Pregestation	• Alpha diversity not statistically different.

et al. 2021	sectional (2007-2012) Spain	term (Gestational age not informed) (N = 71)	(V1-V2 region)			months	al body mass index and breastfeeding until the third month	●Fine motor (FM) skills explained 4% of beta-diversity variation (weighted unifrac) (p < 0.05).<br>●2 enterotypes: *Firmicutes* dominant (Firm) and *Bacteroides* dominant (Bact) → above-median belonged to the Firm-enterotype, and below-median group belonged to the Bact-enterotype (p < 0.05, odds ratio = 0.27);<br>●Above-median FM: high prevalence of *Bifidobacterium, Collinsella, Coprococcus, Enterococcus, Fusobacterium, Holdemanella, Lactobacillus, Propionibacterium, Roseburia, Veillonella*.<br>●Below-median FM: enriched in *Parabacteroides* and *Turicibacter*.
Loughman *et al.* 2020	Longitudinal (Year not informed) Australia	Age > 32 weeks (included pre- and term) (N = 201)	16S rRNA (V4 region)	1, 6, and 12 months of age	CBCL	At 1, 6, and 12 months<br><br>2 years	Gestational age, mode of birth, antibiotic, breastfeeding at four weeks, siblings, household pet. Addicional adjustment: Sex and child's age.	●One- and six-months microbiome showed no association.<br>●At 1 year: decreased *Prevotella* (logFC= -1.46, q < 0.001) and increased *Lachnospiraceae* (logFC=2.09, q = 0.054) in the case group; attenuated when adjusting for confounding (q = 0.1).<br>●For *Lachnospiraceae*, q-values were 0.15, 0.01, and 0.01 respectively for 1-,6-, and 12- month infant temperament.<br>●*Prevotella* at 12 months and two-year behavior outcomes was primarily related to the Internalizing subscale.<br>●Lower *Prevotella* and increased risk of being a case (Internalizing subscale) - adjusted (?). Antibiotics exposure was better predictor of this absence.

								• No assoc. between fecal SCFA and child behavior.
Aatsinki *et al.* 2022	Nested case-control (Year not informed) Finland	Included pre- and term infants (N = 131)	16S rRNA (V4 region)	2.5 months	Eye Tracking of Emotional Attention	8 months	Gestational age, delivery mode, breastfeeding, infant age at sampling, maternal depressive symptoms, and sex	• Alpha and beta diversity not associated with fear or face bias.<br>• Abundance of *Lactobacillus* (log2FC= -6.5), *Bifidobacterium* (log2FC= -5.3), *Prevotella* (log2FC= -5.7), and *Hemophilius* (log2FC= -6.8) were inversely and *Clostridium* (log2FC= 5.4) directly associated with fear bias. All FDR < 0.001.<br>• Fear bias did not show sex interaction with genera.<br>• Random forest model showed *Bifidobacterium, Actinomyces, Clostridium, Collinsella*, and *Parabacteroides* as the most important features related to fear bias.
Aatsinki *et al.* 2019	Longitudinal (2013-2016) Finland	Included pre- and term (N = 301)	16S rRNA	2.5 months	(IBQ-R	6 months	Sex, delivery mode, gestational age, infant age at sampling, antibiotic use, breastfeeding at 2.5 months	• Alpha-diversity associated with negative emotionality (β=-0.17, FDR = 0.17) and fear reactivity (β=-0.27, FDR = 0.17).<br>• *Bacteroides*-cluster had highest and *V. dispar*-cluster lowest microbiota richness and diversity (FDR < 10^{-8}).<br>• *Bifidobacterium/Enterobacteriaceae*-community had the highest and *Bacteroides*-cluster the lowest scores in temperament trait of regulation subscales were: high intensity pleasure, cuddliness and duration of orienting (all FDR = 0.20).<br>• *Bacteroides*–cluster was inversely associated with the main dimension of regulation (β=–0.18, FDR = 0.17).<br>• *V. dispar*-cluster was inversely associated with regulation (β=–0.22, FDR = 0.09) and cuddliness

								(β=−0.29, FDR < 0.01).<br>• Surgency was inversely associated with *Atopobium* and directly associated with *Bifidobacterium* and Streptococcus (FDR > 0.2).<br>• *Erwinia* was directly associated with Regulation and Negative emotionality. *Rothia* and *Serratia* were directly associated with Negative emotionality and Fear reactivity. *Peptoniphilus* and *Atopobium* were directly associated with Fear reactivity.
Rothenberg *et al.* 2021	Cross-sectional (2013-2014) China	Age ≥ 37 weeks (N = 46)	16S rRNA (V3–V4 region)	36 months	BSID	36 months	Child's sex, age at BSID-II assessment, breastfeeding, maternal age and education.	• 3 coabundance factors were found for the 25 most abundant taxa.<br>• Alpha diversity measures were not associated with Bayley scores in adjusted regression models (p > 0.16).<br>• The first Coabundance factor (positive loadings for *Faecalibacterium*, *Sutterella*, and *Clostridium* cluster XIVa) was directly associated with Mental Developmental Index (β = 3.9 points, p < 0.05) and Psychomotor Developmental Index (β = 8.6 points, p < 0.01) in adjusted models.
Zhang *et al.* 2021	Case-control (2019-2020) China	Age ≥ 37 weeks (N = 45 [study group] and 32 [control group]).	16S rRNA (V3 region)	Days 1, 3, and 5	ASQ-3	6 months	Not adjusted	• Higher alpha diversity at day one in case infants (diagnosed with asphyxia) than in the control group (p < 0.05). No significant difference at day 3 and 5.<br>• The dominant microorganisms in the case group on day 1 were *Lachnospiraceae* and *Clostridia* on day 1, and only *Clostridia* on day 3. In the control group, *Staphylococcus* dominated on day 3.

								• The communication score of ASQ-3 (6 months) in the case group was inversely correlated with *Lachnospiraceae* (−0.366; $p < 0.05$) and Clostridia (−0.030; $p < 0.05$) on day 1.
Fox *et al.* 2021	Longitudinal (Year not informed) USA	Gestational age not informed (N = 67)	16S rRNA (V3–V4 region)	1–3 weeks, 2, 6, and 12 months	IBQ-R	12 months	Sex and Breastfeeding	• Beta diversity at age 1–3 weeks was associated with surgency/extraversion (R2 = 0.276, p < .01), subscales included approach (R2 = 0.285, p < .01), high-intensity pleasure (R2 = 0.275, p < .01), and smiling/laughter (R2 = 0.273, p < .01).<br>• *Bifidobacterium* and *Lachnospiraceae* abundance at 1–3 weeks of age was directly associated with surgency/extraversion at age 12 months ($q < 0.01$).<br>• *Klebsiella* abundance at 1–3 weeks was inversely associated with surgency/extraversion at 12 months ($q < 0.01$).<br>• 12-month-old microbiota was associated with negative affectivity at 12 months, specifically *Ruminococcus* and an inverse association with *Lactobacillus* ($q < 0.01$).
Zhang *et al.* 2021	Cross-sectional 2014 China	Age ± 39.6 weeks (N = 38)	16S rRNA (V4-V5 region)	3 years	GDI and CBCL	3 years	Sex and Hydroxyl PAHs (OH-PAHs)	• **GDI:** The phyla *Firmicutes, Actinobacteria, Proteobacteria, Tenericutes,* and *Lentisphaerae* were directly correlated with most domain behavior scores of the GDI.<br>• *Firmicutes* showed a significant correlation with Gross motor behavior score ($r = 0.327$, $p < 0.05$).<br>• *Bacteroidetes*, showed an inverse correlation with the Gross motor score ($r = -0.416$, $p < 0.01$) and *Fusobacteria* with the Adaptive score ($r = -0.334$, $p < 0.05$); and an inverse association with OH-PAH

								levels.<br>● **CBCL:** The phyla *Bacteroidetes, Actinobacteria*, and *Fusobacteria* showed direct correlations with most CBCL core and broadband syndromes. While *Firmicutes, Verrucomicrobia, Synergistetes, Proteobacteria* and *Tenericutes* were inversely correlated.
Kelsey *et al.* 2021	Cross-sectional (Year not informed) USA	Age ± 39.43 weeks (N = 63)	Shotgun metagenomic sequencing	25 days	rs-fNIRS and IBQ-R	25 days	<u>For Shannon:</u> BW, income, breastfeeding, gestational age, head circumference. <u>For functional term diversity:</u> income, gestational age, antibiotics, sex, head circumference. <u>For psychologic</u>	● Direct association between taxa diversity and the left fronto-parietal network (FDR < 0.07) and, alpha diversity and homologous-interhemispheric network connectivity (Chao1-taxa β = 0.16, FDR = 0.10, R2 = 0.07; Shannon-Taxa β = 0.06, FDR = 0.20, R2 = 0.09).<br>● Increased microbial genes encoding for virulence factors linked to increased homologous-interhemispheric connectivity.<br>● **Functional connectivity: 1) Left default mode network:** <u>Group w/ high connectivity</u>: increased *C. perfringens*; **2) Left fronto-parietal network:** <u>Group w/ high connectivity</u>: increased *Enterococcus faecalis, Collinsella, C. disporicum, Prevotella copri, C. perfringens, C. tertium, Robinsoniella peoriensis, C.* Unclass, *Bacteroides* caccae, *E. coli*; <u>decreased:</u> *S. salivarius, Enterococcus Unclass*. **3) Homologous-interhemispheric network:** <u>Group w/ high connectivity</u>: increased *E. coli* and decreased *B. dentium.*<br>● **Temperament: High negative emotionality and regulation/orienting:** increased *B.*

							al outcomes: infant age and income.	*pseudocatenulatum.*
Eckermann *et al.* 2022	Longitudinal (Year not informed) Netherlands	Age ≥37 weeks (N = 156)	16S rRNA (V4 region)	At month 1, 3 and 4 as well as at 6 and 10 years.	BRIEF	8 and 10 years	Breastfeeding, maternal education, child sex and age.	• Any association between fecal microbiota composition and executive function was found (using Random Forest regression, Bayesian linear models and Partitioning around Medoids).<br>• Evaluated only alpha diversity.
Laue *et al.* 2021	Longitudinal (Year not informed) USA	Age ± 39.5 weeks (N = 260)	16S rRNA (V4-V5 region) Shotgun metagenomic sequencing	16S rRNA (6 weeks, 1, and 2 years) N = 523 Shotgun (6 weeks and 1 year) N = 234	BASC-2	3 years	Child sex, gestational age, maternal education, parity, delivery mode, maternal and paternal age at delivery, maternal smoking during pregnancy, early life exclusive breastfeeding, duration	• At 6 weeks, alpha diversity was associated with better Anxiety scale ($p < 0.05$) among boys.<br>• At 2 years, alpha diversity was associated with better Social Skills and Adaptive Skills among boys and better Developmental Social Disorders among girls. All $p < 0.01$.<br>• No association between beta diversity and BASC-2 scores.<br>• **Using 16S:** At 6 weeks: *Bifidobacterium*, *Bacteroides vulgatus*, and a *Streptococcus* were related to better Adaptive Skills scores among boys (FDR < 0.1); *Klebsiella*, *Clostridium*, and *Haemophilus* were related to worse Adaptive Skills among boys (FDR < 0.1); and *Tyzzerella nexilis* was associated with better Depression scores among boys ($q = 0.07$).<br>• At 1 year, *Faecalitalea* was associated with worse Hyperactivity scores, no discernable sex-specific effects ($q = 0.05$).<br>• At 2 years, *Blautia* was associated with worse measures of hyperactivity (FDR < 0.1).

							of any breastfeeding, and child age at follow-up	• **Using Metagenomic sequencing:** At 6 weeks, *Klebsiella oxytoca* was inversely associated with Adaptive Skills (q = 0.02) and Developmental Social Disorders (q = 0.1) scores among boys and *Granulicatella* was associated with worse Anxiety scores among girls (q = 0.05).<br>• At 1 year, *Streptococcus peroris* was associated with lower Depression and Internalizing Problems among girls (q < 0.01).<br>• Several pathways were associated with better Depression scores among boys related to vitamin B6 biosynthesis or NAD salvage.
Wu *et al.* 2021	RCT 2017 China	Age: 7-14 days (66 breast-fed and 133 formula-fed infants)	Analysis of Bifidobacteria (16S high-throughput sequencing)	Baseline, weeks 16 and 24	ASQ-3	Weeks 16 and 24	Maternal education level	Chance to scoring close to the threshold of delay:<br>• 1 domain OR: 0.947 (CI: 0.901–0.996) p < 0.05 (protective for the risk of developmental delay).<br>• ≥ 2 domains 1.001 (0.964–1.039) p = 0.962 (not associated).
Sordillo *et al.* 2019	RCT (Year not informed) USA	Full term (gestational age ± 39.1 weeks) (N = 309)	16S rRNA (V3/V5 regions)	3 to 6 months	ASQ-3	3 years	Gestational age, ethnicity, sex, antibiotic, breastfeeding, c-section, age at ASQ-	• Coabundance scores dominated by *Clostridiales* were associated with poorer ASQ-3 communication (β, –1.12; p = .05) and personal and social (β, –1.44; p = .01) scores.<br>• The *Bacteroides*-dominated coabundance grouping was associated with poorer fine motor scores (β, –2.42; p = .01).

								3, maternal education and age, marital status, income, and clinical site	

GMDS-R: Griffiths Mental Development Scale; MRI: Magnetic Resonance Imaging; ASQ-3: Ages and Stages Questionnaire; BSID: Bayley Scale of Infant Development; MS: Mullen Scales; CBCL: Child Behavior Checklist; IBQ-R: Maternal reports of Infant Behavior Questionnaire -Revised; GDI: Gesell Development Inventory; rs-fNIRS: Resting state functional near infrared spectroscopy; BRIEF: Behavior Rating Inventory of Executive Function.
